# Personalized Treatment Response Assessment for Rare Childhood Tumors Using Microcalorimetry–Exemplified by Use of Carbonic Anhydrase IX and Aquaporin 1 Inhibitors

**DOI:** 10.3390/ijms20204984

**Published:** 2019-10-09

**Authors:** Stephanie J. Gros, Stefan G. Holland-Cunz, Claudiu T. Supuran, Olivier Braissant

**Affiliations:** 1 Department of Pediatric Surgery, University Children’s Hospital Basel, 4031 Basel, Switzerland; stefan.holland-cunz@ukbb.ch; 2 Department Neurofarba, Sezione di Scienze farmaceutiche, University of Florence, 50139 Florence, Italy; claudiu.supuran@unifi.it; 3 Biological Calorimetry Lab, Department of Biomedical Engineering, University of Basel, 4123 Allschwil, Basel, Switzerland; olivier.braissant@unibas.ch

**Keywords:** CAIX inhibitor, AQP1, rare pediatric cancer, isothermal calorimetry, drug susceptibility, clear cell renal carcinoma, organotypic slice culture

## Abstract

We present a novel approach to a personalized therapeutic concept for solid tumors. We illustrate this on a rare childhood tumor for which only a generalized treatment concept exists using carbonic anhydrase IX and aquaporin 1 inhibitors. The use of microcalorimetry as a refined in vitro method for evaluation of drug susceptibility in organotypic slice culture has not previously been established. Rapid microcalorimetric drug response assessment can refine a general treatment concept when it is applied in cases in which tumors do not respond to conventional chemo-radiation treatment. For solid tumors, which do not respond to classical treatment, and especially for rare tumors without an established protocol rapid microcalorimetric drug response testing presents an elegant novel approach to test alternative therapeutic approaches. While improved treatment concepts have led to improved outcome over the past decades, the prognosis of high risk disease is still poor and rethinking of clinical trial design is necessary. A small patient population combined with the necessity to assess experimental therapies for rare solid tumors rather at the time of diagnosis than in relapsed or refractory patients provides great challenges. The possibility to rapidly compare established protocols with innovative therapeutics presents an elegant novel approach to refine and personalize treatment.

## 1. Introduction

Isothermal microcalorimetry allows real-time monitoring of metabolic activity of living organisms. In calorimetric assays the heat production rate is measured. This method is label free and experimental conditions can be widely adapted to allow investigation of different aggregates, probes and organisms. Measurements can also be performed under different oxygen tensions [1]. Isothermal microcalorimetry has recently been described for detection of bacterial infection [2], drug susceptibility testing and drug screening in microbiology [3] and food microbiology [4] as well material testing [5], monitoring, and parasitological applications [6]. Isothermal microcalorimetry is designed for measurements in the range of microwatt, or lower, under essentially isothermal conditions [1].

Isothermal microcalorimetry rather than conventional methods such as enzymatic assays or spectrophotometry can detect the metabolic heat rate of bacteria, protozoan, and hepatocytes of as low as 10^4^–10^5^, 10^3^–10^4^, and 10^2^–10^3^ respectively [6,7]. There are multi-channel microcalorimeters available that can simultaneously measure various samples. This makes it an attractive method to monitor metabolic activity in the biomedical as well as in the pharmaceutical context. The well plate format of specific instruments makes it easily applicable for biological scientists [1]. Microcalorimetric technique is highly sensitive, especially compared to conventional methods, it is label-free and allows for a high through-put experiments [8]. The reaction volume is dependent on the instrument used, but the well plate format can be used to examine all sorts of probes including but not limited to parasites, microbes, bacteria, microtissues, or tissue biopsies [8].

Use of isothermal microcalorimetry measurements to evaluate treatment response in tumor slice cultures has not been described. 

Clear cell renal sarcoma is an extremely rare disease in childhood. Only 7% of childhood cancers originate from the kidney and of these, clear cell renal sarcoma comprises only 2–3% [9,10]. All kidney tumors are treated according to the SIOP 2001 protocol which will be followed by the Umbrella 2016 trial. Previous results of the SIOP 2001 study showed a 5-year event free survival of 79% and an overall survival of 86% for clear cell sarcoma of the kidney. Stage IV disease and young age represented significant adverse prognostic factors [11,12] leading to advancement of disease or relapse. Especially in advanced disease, this treatment regime of traditional chemotherapeutic agents against clear cell sarcoma of the kidney is reaching its limits. Experimental targets include the Sonic Hedgehog signalling pathway (SMO/GLI1 inhibitors), the PI3KeAkt signaling pathway (PI3K inhibitors, Akt inhibitors, mTOR inhibitors), EGFR (erlotinib/gefitinib), BCOR (inhibition of BCOR), demethylating agents (e.g., decitabine), or theoretically the manipulation of the only recurring YWHAE-NUTM2B/E fusion transcript, but so far controlled clinical data are missing [13,14].

While the treatment concept of clear cell renal carcinoma lacks refinement, all advanced solid tumors have, at some point, the limitation of conventional radio-chemotherapy in common. The therapeutic principle tested here, is inhibition of proteins regulated by the HIF1α pathway and upregulated in hypoxia. We have previously studied the role of several hypoxia-dependent factors in esophageal carcinoma and neuroblastoma [15,16], two tumor entities with poor prognosis in advanced stages of disease. Adaptation of cancer cells to the hypoxic microenvironment is characterized by genetic and adaptive mechanisms allowing the cells to survive. Key regulators in this response to hypoxia are mediated by hypoxia inducible factors, namely HIF-1α und HIF-2α [17]. During this adaptation the hypoxic cancer cells acquire invasive and metastatic properties as well as resistance to chemotherapy and radiation therapy. The hypoxia-dependent enzyme carbonic anhydrase IX (CAIX), amongst others, has been shown to be involved in numerous pathological processes including tumorgenicity, specifically invasion, advanced disease, tumor progression and poorer survival in several other solid tumors [18]. We have previously investigated the role of the hypoxia-dependent enzyme CAIX, which is modulated by HIF-1α and up-regulated in the hypoxic microenvironment, in esophageal carcinoma and neuroblastoma. The use of specific CAIX inhibitors correlated with an anti-proliferative response in cell culture experiments [15,16]. Expression of aquaporin 1 (AQP1), the first molecular water channel described [19], has been reported to be associated with a poor prognosis, especially in later stages of several solid tumors [20], when one can assume that hypoxia within the tumor is increasing. This would suggest that AQP1 is regulated by mechanisms within the hypoxic tumor microenvironment. There is evidence that up-regulation of AQP1 expression is linked to hypoxia [21].

The use of organotypic tumor slice cultures allows us to mimick and influence the tumor microenvironment of a solid tumor in vitro. Moreover the implementation of microcalorimetric measurement of drug response in organotypic tumor slice cultures equips us to test for novel additive treatment strategies in the concept of personalized medicine.

## 2. Results

**Figure 1:**
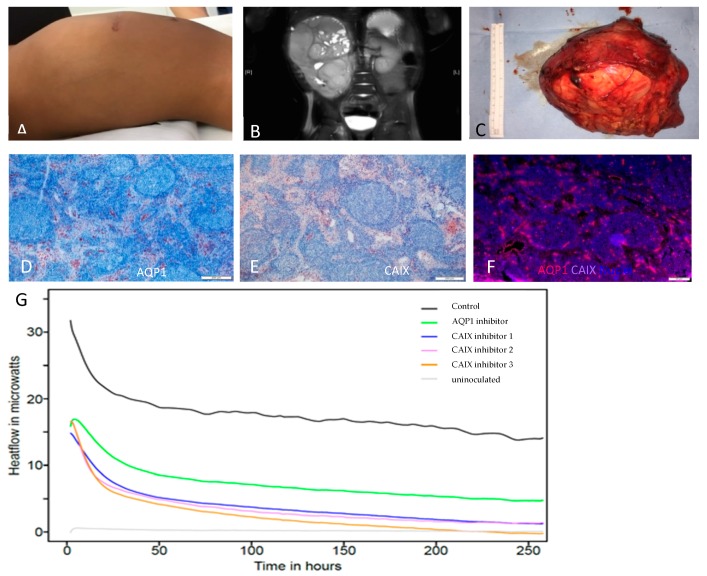
Clinical presentation (**A**) The child presented with a large abdominal mass, which was not reduced in size after initial chemotherapy. The scar was a result of the initial surgical biopsy. (**B**) MRI of primary clear cell sarcoma of left kidney following chemotherapy. Despite few necrotic areas the tumor is largely intact and appears to be vital. (**C**) Large tumor after resection. Immuno-staining (**D** AQP1 IHC, **E**: CAIX IHC, **F**: AQP1 and CAIX IF stainings) The response to the specific inhibitors corresponded with the protein expression profiles of AQP1 (mostly in vibrovascular septae and vessels and CAIX (most strongly within tumor nests). Microcalorimteric measurements (**G**) Microcalorimetric measurements showed a decrease metabolic activity under treatment with AQP and CAIX inhibitors compared to medium control. (Control – medium, Drug 1—AQP1 inhibitor TEA, Drug 2—FC8-325A, Drug 3—FC8-207A, Drug 4—FC12-520A).

Here we show the example of a patient who presented with stage IV clear cell sarcoma of the kidney at 18 months of age. The patient was treated according to the SIOP 2001 protocol and showed stable disease after three month of treatment with a persistently large abdominal mass originating from the right kidney on clinical examination (A), in MRI (B) and after resection (C) (Figure 1A–C). In order to reduce the overall tumor burden of the patient a non-curative resection of the primary tumor was performed. Tumor tissue was examined using immuno-histochemical analysis of AQP1 and CAIX and treatment response assessment using isothermal microcalorimetry of tissue slice cultures.

In the untreated tumor CAIX was expressed almost ubiquitously in all cells of tumor nests and stromal components of the renal clear cell sarcoma (Figure 1 D AQP1 IHC, E: CAIX IHC, F: AQP1 and CAIX IF stainings). Immunostaining showed that AQP1 was mostly expressed in the cells of the fibrovascular septae, but not in the tumor cell nests. AQP1 positive staining of microvessels in the tissue served as positive control. Tumor slice tissues could successfully be cultured over several days and control tissues were still producing an active heat flow at the termination of the experiment after 250 h (Figure 1G). The organotypic tumor slices grown in slice culture without treatment showed viable tissue after five days (data not shown). Using microcalorimetric assessment the heat flow of slice cultures could be significantly modulated by treatment with specific inhibitors of the target enzyme CAIX using three different substances. The CAIX inhibitor FC12-520A gave the strongest heat decrease response. To a lesser extent, but still impressively, the AQP1 inhibitor TEA decreased heat flow of the slice culture. This was consistent in all samples within the treatment groups. No bacterial contamination was observed, thus excluding other sources of metabolic activity but heat production from the tumor slices. We find here that the decrease of the tumor heat production is faster the higher the respective marker is expressed, suggesting that CAIX inhibition is more successful than AQP1 inhibition in this case. Although FC12-520A leads to the strongest response amongst the CAIX inhibitors all CAIX inhibitors show a heat flow reduction in a similar range, demonstrating consistency of CAIX inhibition. 

## 3. Discussion

The patient presented with stage IV disease at an age of 18 months. Stage IV disease and age are the only two known significantly adverse prognostic factors for this disease [11,12]. The patient was treated according to the SIOP 2001 protocol, a treatment protocol used for all pediatric renal tumors, and after three month of treatment showed stable disease (Figure 1A–C). The interdisciplinary tumor board in agreement with a study director representative decided to perform a non-curative resection of the primary tumor to reduce tumor burden. This history is representative for children with rare childhood tumors. There are numerous tumors that are so rare that, although treatment in many countries is already confined to specialized centers, oncologists, radiotherapists and surgeons lack experience in dealing with these tumors. Histological examination of the resected specimen confirmed the poor response to the administered chemotherapy with a tumor necrosis rate of only up to 25% in a very large tumor mass. 

Cancer cells are characterized by dysregulated cell proliferation, and the blood vessels that form within solid tumors are often structurally and functionally abnormal, resulting in severe hypoxia [17]. Regulation of the enzyme carbonic anhydrase IX as well as probably AQP1 by HIF-1α furthers tumor progression and poorer survival in several other solid tumors [18,21,22]. Boros et al. have proposed that mechanisms similar to the inhibition of carbonic anhydrase can inhibit growth of tumor cells by limiting uptake of deuterated water into cells [23,24]. These authors further suggest that these mechanisms might result in metabolic changes of translational impact [25]. The particular carbonic anhydrase IX inhibitors used in our experiment were chosen because they have previously proven to be highly effective in cell culture studies on esophageal carcinoma and neuroblastoma, which like clear cell renal sarcoma represents a rare solid childhood tumor and is classified as rare disease [15,16]. Just as AQP1 inhibitors they target factors that are linked to the hypoxic expression profile of the growing tumors microenvironment. 

The use of microcalorimetry as a refined in vitro method for evaluation of drug susceptibility in organotypic slice culture has not previously been established. With our experiments we demonstrate that within 48 h we are able to detect a response to anti-tumor substances by using microcalorimetric measurements of tumor slice cultures alone. The main advantage of using tumor slice culture for evaluation lies in the possibility of using any tumor piece that is resected during an operation without having to go through the long lasting tasks of obtaining a stable tumor cell culture first. This approach allows for testing of novel therapeutic approaches against standard chemotherapeutic agents in the future. At the same time microcalorimetry represents a reproducible, stable and fast method to test drug response for several drugs at the same time. It will give important insights on the impact of the novel therapeutic on the individual patient’s tumor, facilitating the transition to a clinical study and towards personalized tumor treatment.

Clear cell renal sarcoma often presents with nests of cells separated by fibrovascular septae [12], that we also see in our patient sample, constituting a defined tumor microenvironment. Several methods have been applied to investigate tumors of individuals in an approach to personalize treatment including 2D culture of dissociated tumor cells, 3D culture of spheroid tumors [26], patient derived mouse xenografts [27] and organotypic tumor slice cultures [28]. Of these, the organotypic tumor slice culture is the only method that allows one to study primary culture of an entire tumor piece. This enables one also to investigate the tumor environment e.g., under hypoxia, if only for a short time. Although the organotypic slice culture, as an in vitro method, has been successfully employed for breast cancer and for neuronal studies of the CNS, it has neither been applied to rare childhood tumors nor in combination with microcalorimetry. Slice culture allows for monitoring and visualizing the migration process of cells in an environment that is tissue specific [29]. The use of microcalorimetry as a refined in vitro method for evaluation of drug susceptibility in organotypic slice culture has never been tested. For all solid tumors, which do not respond to classical treatment, and especially for rare tumors without an established protocol this presents an elegant novel approach to test alternative therapeutic approaches. The set-up of current clinic trials allows only for testing of experimental therapies in very high risk patients at very late stages of tumor disease. The fact that most solid tumors in childhood are rare diseases and patients and tumor material are sparse presents an even greater challenge to introduce new therapeutic drugs. Evaluating experimental treatments by using rapid microcalorimetric drug response at the time of diagnosis could present an elegant novel approach to test therapeutic alternatives. The possibility to rapidly compare established protocols with innovative therapeutics would be of great advantage in translating findings from cell based assays to the organotypic slice culture and facilitate translation to human application. Furthermore it could personalize treatment of all solid tumors and make it more effective for each patient at time of diagnosis and of disease relaps.

## 4. Materials and Methods

### 4.1. Tumor Slice Culture and Microcalorimetric Measurement

Tumor tissue was taken from the resected specimen immediately after surgery. Written consent was obtained prior to the operation and use of human tissue was in accordance with the ethics approval (EKNZ 2015-263). Tissue slice culture pieces were prepared using scalpel dissection under the binocular. Tissues were taken from the same tumor area, matched regarding their tumor/stroma ratio, they were weight and measured for weight/area correction. Tissue slices were cultured in TUM medium [30] at 37 °C for 24 h. Following this initial incubation period the medium was changed and specific experimental inhibitors (CAIX inhibitors: FC8-325A, FC8-207A, FC12-520A and AQP1 inhibitor TEA (Merck, Sigma-Aldrich Chemie GmbH Buchs, Switzerland) added to final concentrations of 500 μM [15,16]. Control samples containing TUM medium were included. Each treatment group, including the control group, contained four tumor samples (*n* = 4/group). Separate control samples were grown in slice culture in TUM medium and evaluated for tissue viability.

### 4.2. Isothermal Microcalorimetry

For microcalorimetric measurements a pre-production model of a 48-channel isothermal microcalorimeter (Symcel Sverige AB, Spanga, Sweden) was used as previously described [6]. Tumor slice culture pieces were weighed and measured for weight/area correction. They were transferred to the vials containing experimental inhibitors or control TUM medium. The vials were then sealed and inserted in the well-plate microcalorimeter according to manufacturer instructions. One position in the plate was charged with an inert sample, which was used as a reference. For optimal performance multiple separate reference vessels were included. Each reference vessel was filled with an inert sample (medium only), which was used as a thermal reference. Following thermal equilibration measurements were recorded with the thermostat set at 37 °C. The microcalorimeter data were sampled at a frequency of 1 data point every 60 s over >250 h until the metabolic heat signal returned to baseline. Data were stored by the Symcel Calview software and exported as a CVS file that could be edited in commonly used spreadsheet software. Finally 10 µL of the culture medium were streaked on a brain heart infusion (BHI) agar plate to ensure the absence of bacterial contamination. 

### 4.3. Immunostaining

Tumor sample was snap frozen, cut to a thickness of 7 μm and mounted onto glas slides. The HRP-AEC (R&D Systems, Mineapolis, MN, USA) was used for the staining. AQP1 and CAIX staining was performed according to the protocol using a primary polyclonal rabbit anti-AQP1 antibody (Merck Millipore, Sigma-Aldrich Chemie GmbH Buchs, Switzerland) at a dilution of 1:400 and antibody M75 (BioScience Slovakia, Bratislava, Slovak republic) at a dilution of 1:200. Counterstaining was achieved with hematoxilin solution (Spitalpharmazie USB Basel, Switzerland). For immunofluorescence staining slide were fixed and permeabilized with 4% paraformaldehyde in phosphate buffered solution. Blocking was done with 3% bovine serum albumine in phosphate buffered solution with Tween 20 for one hour at room temperature. Primary antibodies M75 and anti AQP1 were used at the same conditions as above. Detection was done with Goat anti-Mouse IgG (H+L) Cross-Adsorbed Secondary Antibody, Alexa Fluor 647 and Goat anti-Rabbit IgG (H+L) Cross-Adsorbed Secondary Antibody, Alexa Fluor 555 (both Invitrogen, Thermo Fisher Scientific Inc., Waltham, MA, USA) respectively. ProLong^®^ Gold Antifade Mountant with DAPI (Life Technologies, Thermo Fisher Scientific Inc., Waltham, MA, USA) was used for DNA staining and mounting. Control sections were incubated with secondary antibody control. Imaging was performed on an Olympus BX43 microscope using CellSens software.

## Abbreviations

CAIX	Carbonic anhydrase IX
AQP1	Aquaporin 1
